# Bioinspired design of flexible armor based on chiton scales

**DOI:** 10.1038/s41467-019-13215-0

**Published:** 2019-12-10

**Authors:** Matthew Connors, Ting Yang, Ahmed Hosny, Zhifei Deng, Fatemeh Yazdandoost, Hajar Massaadi, Douglas Eernisse, Reza Mirzaeifar, Mason N. Dean, James C. Weaver, Christine Ortiz, Ling Li

**Affiliations:** 1 Department of Materials Science and Engineering, Massachusetts Institute of Technology, Cambridge, MA 02139-4307 USA; 2 Department of Mechanical Engineering, Virginia Polytechnic Institute and State University, Blacksburg, VA 24060 USA; 3 Department of Radiation Oncology, Dana-Farber Cancer Institute, Harvard Medical School, Boston, MA 02115 USA; 4 Department of Biological Science, California State University Fullerton, Fullerton, CA 92834 USA; 5 Department of Biomaterials, Max Planck Institute of Colloids and Interfaces, Am Muehlenberg 1, 14424 Potsdam, Germany; 6 Wyss Institute for Biologically Inspired Engineering, Harvard University, Cambridge, MA 02138 USA

**Keywords:** Bioinspired materials, Mechanical properties

## Abstract

Man-made armors often rely on rigid structures for mechanical protection, which typically results in a trade-off with flexibility and maneuverability. Chitons, a group of marine mollusks, evolved scaled armors that address similar challenges. Many chiton species possess hundreds of small, mineralized scales arrayed on the soft girdle that surrounds their overlapping shell plates. Ensuring both flexibility for locomotion and protection of the underlying soft body, the scaled girdle is an excellent model for multifunctional armor design. Here we conduct a systematic study of the material composition, nanomechanical properties, three-dimensional geometry, and interspecific structural diversity of chiton girdle scales. Moreover, inspired by the tessellated organization of chiton scales, we fabricate a synthetic flexible scaled armor analogue using parametric computational modeling and multi-material 3D printing. This approach allows us to conduct a quantitative evaluation of our chiton-inspired armor to assess its orientation-dependent flexibility and protection capabilities.

## Abstract (web summary)

Biology has often served as the inspiration for the design of body armor; one common limitation is the flexibility of the resultant armor. Here, the authors examine the armour of chiton and use the observed design principles to 3D print flexible armor.

## Introduction

A primary function of biological armors is to provide mechanical protection from the surrounding environment, including attacks from potential predators^1–6^. Over millions of years, a variety of forms of biological armors have evolved, ranging from the hard shells of mollusks^7^ to the thick helmet-like skulls of the pachycephalosaurids^8^. Many of these biological armors are often multifunctional, simultaneously offering mechanical protection and performing other functional roles such as hydrodynamic drag reduction^9^, coloration or optical sensing^10,11^, chemo- or mechano-sensing^12^, and vision^13^. Flexibility, however, is a more challenging function to pair with protection, since most biological armors are based on rigid and hard armor plates. A solution to maximizing both of these seemingly opposing performance metrics is the development of scale-like armors consisting of small, repeating elements (rather than a few larger ones), as are found in familiar vertebrates such as cartilaginous and bony fishes, turtles, armadillos, crocodiles, and pangolins^5^.

Scale-based biological armors can be classified into two categories, overlapping scales (e.g., in pangolins, some fish and snakes) and abutting bony plates, also known as osteoderms (e.g., in turtles, lizards, alligators, and armadillos)^5^. The former category, particularly fish scales, includes many popular model systems for investigating the dual protection-flexibility performance of scaled armors^5^, which results from scales’ rotating, sliding and bending relative to each other^14–17^. In contrast to fish scales, the flexibility of osteoderm-based armors results from the relative motion of adjacent plates, either through their connecting elastic Sharpey’s fibers (e.g., armadillo scales^18^) or interdigitating sutures (e.g., in turtles)^19–21^. The flexibility of these osteoderm-based armors can also vary significantly depending on the structural complexity and degree of structural interdigitation. For example, the maximum bending angles of red-eared and leatherback turtle shell plates are ca. 1 and 15 degrees, respectively, a function of morphological differences in the interlocking structures of adjacent osteoderms^20,21^. At a far smaller scale, sharks and rays utilize a similar protective strategy for their cartilaginous skeletons, which are covered with tiled arrays of sub-millimeter-sized plate-like tiles called tesserae. The tessarae allow both mechanical protection and flexibility by behaving differently when the skeleton is loaded in tension or compression^22–25^.

**Fig. 1:**
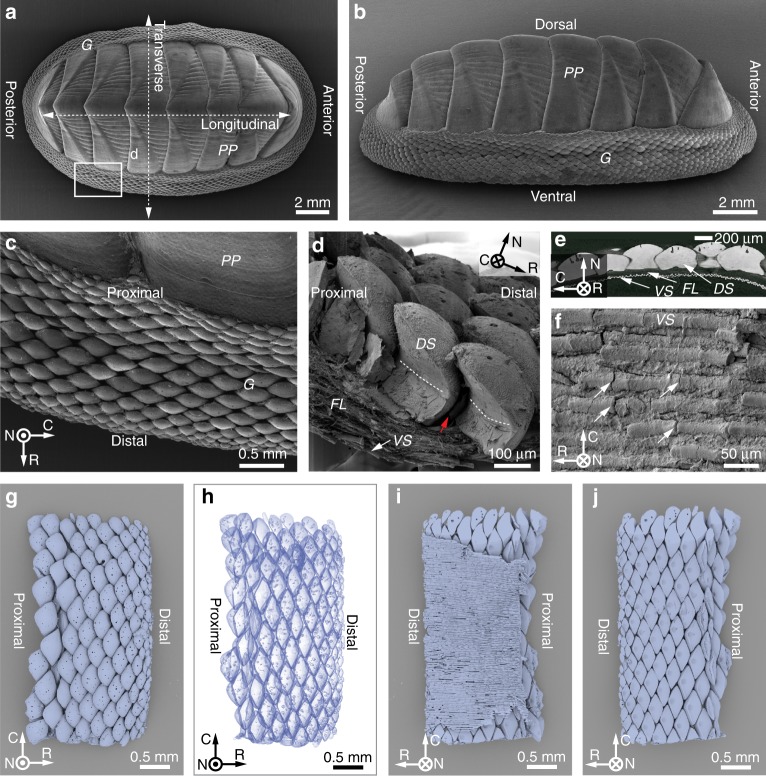
Biological flexible scaled armor in the girdle of the chiton *Rhyssoplax canariensis*. **a**, **b** Wide-field scanning electron microscopy (SEM) images of the chiton *R. canariensis*, which show the dorsal and side view of the primary plates (PP) and peripheral scale-covered girdle (G), respectively. **c** Enlarged view of the girdle covered with dorsal scales. The image was acquired from the region indicated by the rectangular box in **a**, highlighting individual overlapping dorsal girdle scales, a fully covered protective armor. **d** Fractured cross-section of the girdle scaled armor, which consists of three components arranged from dorsal to ventral: (1) dorsal scales (*DS*); (2) fibrous layer (*FL*); and (3) ventral scales (*VS*). The white dashed lines indicate the height of the inter-scale organic matrix, and the red arrow indicates gaps between adjacent scales. **e** Cross-sectional view of scaled armor based on micro-computed tomography (*μ-CT*) data. Note the distance between the dorsal and ventral scale layers, which is occupied by the fibrous layer. **f** SEM image of the rod-shaped ventral scales, where the white arrows indicate small cracks. **g**–**i** *μ-CT* 3D rendering of the chiton *R. canariensis* girdles in different viewing orientations and modes: **g** top view of girdle scales, **h** transparency mode showing the overlapping characteristics among adjacent scales, bottom view **i** with and **j** without ventral scales. A local coordinate system is defined as following: *N* (normal), from ventral to dorsal; *R* (radial), from proximal to distal; *C* (circumferential).

The combination of protection and flexibility, which has evolved multiple times in biological armors, has challenged designers of human body armors throughout human history. Man-made examples range from the highly protective but cumbersome Medieval armors, to the more flexible and lightweight Japanese Samurai armors, to recent advancements in the development of bio-inspired flexible protection systems^16,26,27^. In these recent efforts, parametric modeling and three-dimensional (3D) printing has become a powerful means to investigate the mechanical behavior of both biological and bio-inspired materials and structures^15,16,28–33^. While most previous research on biological flexible armors focused on the study of fish scales (which consist of a mineralized collagen-based plywood-like structure) or osteoderms (porous bony plates), there are many other specialized biological armors worthy of investigation. In the present study, we report one such example from an ancient lineage of mollusks known as the chitons. In contrast to most shelled mollusks where mobility is limited, as in the single shelled mollusks (gastropods, including snails, scaphopods or tusk shells, and some cephalopods such as *Nautilus*) or hinge-shelled bivalves (mussels, clams, scallops, etc.), most polyplacophorans (chitons) are characterized by eight overlapping, hard shell plates (Fig. 1a, b), which collectively accommodate a wide range of motion^34–36^. In addition to the eight overlapping shell plates (which are functionally analogous to the segmented plate-like exoskeleton of many crustaceans), additional protection is provided by a thick leathery girdle that skirts the animal’s periphery^37^ (Fig. 1a, b). The combined maneuverability of the shell plates and the girdle facilitates locomotion and conformation to uneven surfaces, as well as protection from potential predators^38^ and desiccation prevention when the animals are exposed at low tide.

In one lineage of chitons (Chitonina), the girdles of many genera are completely covered with small, overlapping scales (Fig. 1a–c). These scales are unlike fish scales, osteoderms, or tesserae, in that they are almost pure mineral (lacking any significant quantities of organic material). Despite the rigidity of individual scales, the scaled girdles are extremely flexible and capable of conforming to rough surfaces^39,40^, while also locally wrinkling along the margins to form inhalant and exhalent channels for the circulation of seawater^35,41,42^. The degree of girdle flexibility is remarkable considering the scales are composed of ca. 97% aragonite by weight^43^, densely packed, and often exhibit appreciable overlap^44–47^. The scale surface can be smooth or intricately ribbed. These fine details of the dorsal surface of the scales have long been used to distinguish species, with taxonomic accounts featuring detailed hand-drawn illustrations^48–50^, light micrographs^51^, or scanning electron micrographs^37^. Despite their frequent use for species identification purposes, very little is known regarding the scales’ material composition, mechanical properties, three-dimensional morphology, large-scale organization, or biomechanical and protective properties in the context of flexible armor design.

The present study provides a comprehensive investigation of the aforementioned aspects of chiton scales through a wide range of experimental and modeling approaches, including electron microscopy, instrumented nanoindentation, synchrotron X-ray micro-computed tomography, mechanical testing, and finite element modeling. Incorporating the physical and functional properties of chiton girdle scales characterized in these investigations, we design a bio-inspired flexible armor system, integrating parametric geometrical modeling and multi-material 3D printing. We explore the functional trade-offs between protection and flexibility in this model scaled armor system and its potential for informing the design of additional functional prototypes.

## Results

### Morphological, compositional, and mechanical properties

The chiton *Rhyssoplax canariensis* (Chitonidae: Chitoninae) was chosen as a representative model system to conduct a detailed investigation of chiton girdle scale armors. Like other chitons, this species (ca. 20 mm in length) is covered by eight bilaterally symmetrical overlapping mineralized shell plates, which are arranged dorsally along the longitudinal axis (in an anterior-posterior direction, Fig. 1a, b). In addition to the eight primary shell plates, protection is provided by the scaled girdle, which runs along the perimeter of the entire body (Fig. 1a, b). The girdle scales (ranging in size from ca. 100 μm to ca. 500 μm) are tightly packed and extend from the primary shell plates to the girdle edge (Fig. 1c). These tiny scales cover the girdle completely, and no gaps are observed between the primary plates and girdle scales when viewed dorsally. In this work, a pseudo-cylindrical coordinate system was used for denoting the sample orientations, where *N*, *R,* and *C* refer, respectively, to the normal (dorsal), radial, and circumferential directions at a given point along the girdle.

**Fig. 2:**
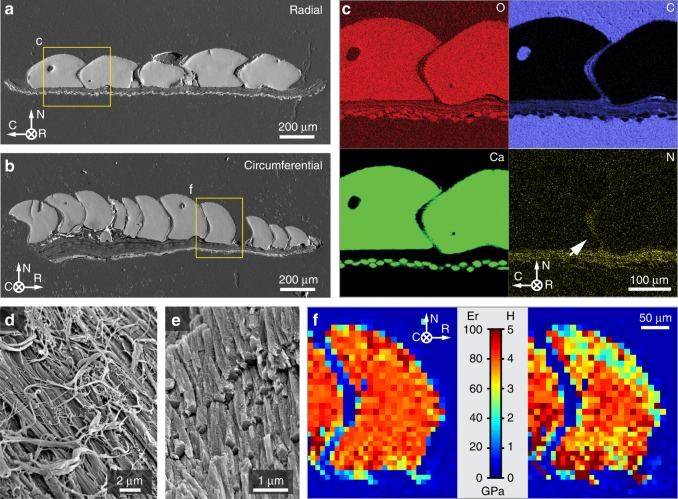
Compositional and mechanical characteristics of the girdle scales in the chiton *R. canariensis*. Backscattered SEM images of polished cross sections of the chiton’s scaled girdles in two orientations: **a** radial and **b** circumferential. Note that the middle organic fibrous layer can be clearly distinguished from the dorsal and ventral scales due to its electron density difference. **c** Energy-dispersive X-ray (EDX) spectroscopic elemental maps (O, C, Ca, and N) of the scale assembly taken from the region shown in **a**. The white arrow indicates the presence of nitrogen between adjacent scales above the fibrous middle layer. SEM image of the **d** fibrous middle layer after dorsal scale removal, and **e** the rod-like crystals observed on the fracture surface of an individual dorsal scale. **f** Indentation moduli, *E*_*r*_, and hardness, *H*, maps of the chiton scales taken in the region shown in **b** obtained from instrumented indentation measurements.

When viewed in cross-section, the girdle has three main structural components: the layer of large dorsal girdle scales (upper), a fibrous layer (middle), and a layer of smaller ventral scales (lower) (Fig. 1d, e). The dorsal scales exhibit a hook-like geometry with curvature towards the body, and while gaps between adjacent dorsal scales can be easily seen in sectioned or torn samples (red arrow, Fig. 1d),  which are not externally visible due to the overlapping of individual scales (Fig. 1c). The bases of these dorsal scales are directly attached to the underlying fibrous layer (Fig. 1d). The white dashed lines in Fig. 1d, located close to the height at which the overlapping dorsal hook begins to form, suggest possible locations up to which the scales were covered by inter-scale soft tissues (also see Fig. 2c). The presence of this inter-scale organic matrix was also supported by the fact that after mechanically removing the underlying fibrous layer, the integrity of the dorsal girdle scales assembly was maintained (Supplementary Fig. 1). In contrast to the larger dorsal scales, the ventral scales are smaller and characterized by a comparatively simpler rod-like morphology (diameter, ca. 20 μm; length, 100–300 μm), with their longitudinal axes oriented in the radial direction from the animal’s geometric center (Fig. 1f).

Synchrotron X-ray micro-computed tomography (*μ-CT*) was used to further investigate the three-dimensional geometry of the girdle scale assembly (Fig. 1g–j). The highly X-ray absorbent mineralized scales were easily segmented from the non-mineralized fibrous layer and inter-scale organic matrix. The dorsal girdle scales of *R. canariensis* are tightly packed despite a gradual decrease in scale size from the proximal to distal margins (Fig. 1g), and the overlap between adjacent scales can be visualized by using the transparent mode of 3D reconstructions (Fig. 1h). The rod-like ventral scales, by contrast, align their longitudinal directions along the radial (proximal-distal) direction, providing a complete and uniform coverage of the ventral surface of the girdle (Fig. 1i). The tight packing of the dorsal scales results in their diamond-shaped bases forming a diamond mosaic pattern when viewed ventrally, as revealed by the reconstructions with their ventral scales removed (Fig. 1j).

Compositional and nanomechanical measurements of the individual components of the girdle armor were then conducted to gain additional insight into its mechanical performance. Backscattered SEM (BSEM) images acquired from polished cross sections of the girdle armor in both radial and circumferential orientations demonstrate the high and uniform electron density of scales relative to the middle fibrous layer (Fig. 2a, b). The spacing between adjacent dorsal scales measures ca. 5 μm throughout the assembly. The circumferential cross-section shows that the thickness of the middle fibrous layer gradually decreases from the chiton body (ca. 200 μm) to the distal margin of the girdle (ca. 10 μm), and accompanies a similar corresponding decrease in dorsal scale sizes. Energy-dispersive X-ray spectroscopy (EDS) measurements confirmed that calcium carbonate is the main constituent in the dorsal and ventral scales, while the fibrous middle layer is not mineralized (Fig. 2c). Nitrogen was detected in the middle layer and in the regions between the scales (white arrow in Fig. 2c, N map), but not in the surrounding epoxy, suggesting the presence of a N-containing inter-scale organic matrix. Higher magnification SEM studies of fractured girdle samples revealed that the constituent fibers (measuring several hundred nm in diameter and >μm in length) of the middle fibrous layer exhibit distinctive band-like striations (Fig. 2d and Supplementary Fig. 2). High-resolution SEM image of fractured dorsal scales revealed an internal microstructure of tightly packed rod-like building blocks (Fig. 2e).

Instrumented nanoindentation measurements showed that the mechanical properties within individual scales were uniform due to the lack of significant sub-layering or structural gradients. The mineralized scales had a reduced modulus of 79.7 ± 4.6 GPa and hardness of 3.8 ± 0.6 GPa (*n* = 96), values which are comparable to those obtained from aragonite-based mineralized structures found in other mollusks^52,53^ (Fig. 2f).

### Three-dimensional geometry

**Fig. 3:**
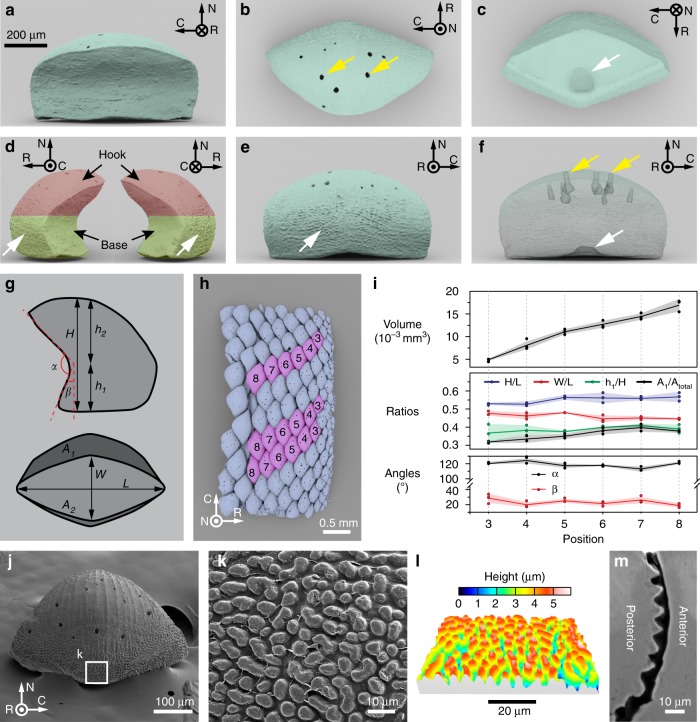
Three-dimensional geometry and surface morphology of individual dorsal scales of the chiton *R. canariensis*. **a–f** μ-CT data-based 3D rendering of individual girdle scales in different view angles and modes: **a** front view, **b** top view (yellow arrows indicate pore openings), **c** bottom view (white arrow shows a depression at the base of the scale), **d** two side-views (white arrows shows the surface roughness at the lower surface of backside), **e** back view, and **f** transparent mode (the yellow arrows show holes in the dorsal surface of scales and the white arrow indicates depression in base). **g** Projection contours along two orientations (transverse and bottom) are used to describe the geometries of chiton scales. Geometrical parameters are defined, including lengths (*w*, *l*, *h*_1_, *h*_2_, *h* (=*h*_1_ + *h*_2_)), angles (*α* and *β*), areas (*A*_1_, *A*_2_, and *A*_total_ (=*A*_1_ + *A*_2_)), and volumes (*V*). **h** Top view of a μ-CT data-based reconstruction of the girdle scale assembly of *R. canariensis*. Three columns of scales used in the geometrical measurement are highlighted in pink color and their positions are indicated. **i** Variations of geometrical parameters as a function of scale position. The solid line represents the average and the shaded area shows the standard deviation (*N* = 3 for each measurement). **j** SEM image of a scale’s back surface. **k** Magnified-view of scale surface with microscopic bumps at the underside of the back surfaces of chiton scales, as indicated by the white box in **j**. **l** SEM-derived stereographic reconstruction of microscopic bumps in a similar region shown in **k**. **m** Backscattered SEM image of a polished cross-section in the region of microscopic bumps of a scale, highlighting the difference in morphology between the anterior (front) and posterior (back) surfaces of the dorsal scales.

The 3D geometry and surface morphology of individual girdle scales from *R. canariensis* based on synchrotron *μ-CT* measurements are summarized in Fig. 3. Three-dimensional reconstructions of individual dorsal scales in different views highlight their two main geometrical features: a diamond-shaped prismatic ventral base and a shallow, cup-like dorsal hook that overlaps with adjacent scales (Fig. 3a–f). Fine structural features include a dimple at the scale base (white arrow in Fig. 3c) and a roughening of the surface of the posterior margin of each scale (white arrows in Fig. 3d, e). Small depressions were observed on the dorsal surface of scales (Fig. 3b), typically extending to a depth of ca. 100 μm, as shown in the transparent reconstruction in Fig. 3f.

In order to successfully mimic scale morphology for the production of a 3D-printed structural analogue (as discussed later), quantitative measurements of the scale geometry were conducted by defining several morphometric parameters, including *W* (width of base), *L* (length of base), *h*_1_ (the vertical height from the base to the inflection point), and *h*_2_ (the vertical height from the inflection point to the top of the scale), imbrication angle *α* (describing the medial lean of the hook), inclination angle *β* (describing the lateral pitch of the base), and two projection areas *A*_1_ and *A*_2_ (Fig. 3g). Morphometric measurements from three rows of dorsal scales within the girdle scale assembly were conducted (Fig. 3h), and the results are summarized in Fig. 3i. The scale size gradually decreases from the proximal to distal margins, as indicated by the decrease of scale volume from ca. 16 × 10^−3^ mm^3^ to ca. 5 × 10^−3^ mm^3^. Despite variations in size, all scales are roughly geometrically similar, as evidenced by the consistency of all parametric ratios (*H/L*, ca. 0.5–0.6; *W/L*, ca. 0.4–0.5; and *h*_1_*/H*, ca. 0.3–0.4), angles (*α*, ca. 110–120° and *β*, ca. 20–30°), and overlapping ratio (*A*_1_*/A*_tota*l*_, ca. 0.3–0.4), where *H* *=* *h*_1_ *+* *h*_2_ and *A*_total_ *=* *A*_1_ *+* *A*_2_.

SEM imaging of isolated individual dorsal scales revealed the presence of evenly spaced ridges (spacing: ca. 40 μm) running along the dorsal surface of the scales proximodistally toward the hook tip (Fig. 3j). The rear view of isolated scales shown in Fig. 3j illustrates the gradual increase in surface roughness towards the base of the scales, consistent with our *μ-CT* measurements (Fig. 3d, e). Figure 3k shows that the roughness of the posterior surface is caused by evenly distributed cone-shaped protrusions, with the corresponding stereographic reconstruction of the surface revealing that both their height and wavelength are ca. 5 μm (Fig. 3l). The surface rugosity of posterior side is in stark contrast to the surface smoothness of the anterior side of scales, when viewed in cross-section (Fig. 3m). Similar surface features were described by Bullock from the genus *Chiton* (also Chitoninae)^54^.

### Interspecific comparison

**Fig. 4:**
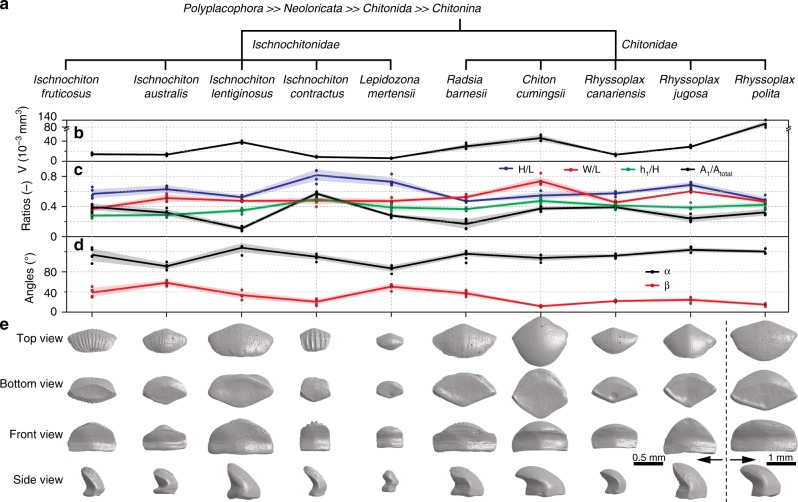
Interspecific comparison of the dorsal scale geometries among ten chiton species. **a** The phylogenetic distribution of the chiton species used in this study. Variations of morphological parameters, **b** volume, **c** length and area ratios, and **d** angles for different species. The solid line represents the average and the shaded area shows the standard deviations (*N* = 5 for each measurement). **e** μ-CT data-based 3D reconstructions of individual dorsal scales from the ten chiton species in four different views: top, bottom, front, and side. All the reconstructions are at the same length scale except those from *Rhyssoplax polita*.

**Fig. 5:**
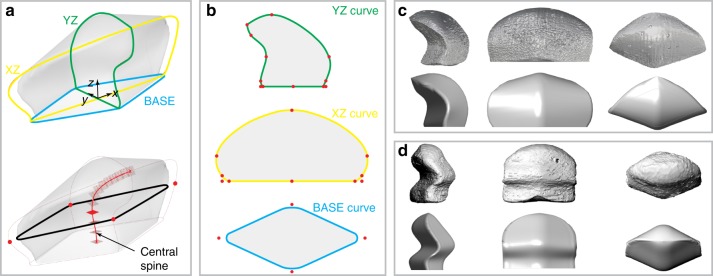
Three-dimensional parametric modeling of chiton scale geometries. **a** Top, the 3D scale model with three principal scaffolding curves, XZ, YZ, and BASE. Bottom, 3D scale model highlighted with the central spine for generating the surface meshes. **b** Three principal curves with geometrical landmarks indicated. **c**, **d** Comparison of the original chiton scales with corresponding mimicked scale models for two species: **c** a single-curved scale from chiton *Rhyssoplax canariensis* and **d** a double-curved scale from chiton *Lepidozona mertensii*.

We extended our 3D morphometric measurements of girdle dorsal scales to multiple chiton species from two families, the *Ischnochitonidae* and the *Chitonidae* (Fig. 4a). Scales were selected from the middle region of the girdles (approximately equidistant from the proximal and distal margins) for consistent geometrical and size comparisons. Top-, bottom-, front-, and side-view images of the different scales are shown in Fig. 4e. Five scales were segmented from the *μ-CT* data obtained from each of the ten species, and their morphometric measurements were recorded (Figs. 3g and 4b–d). The scales exhibited large variations in volume from ca. 0.005 mm^3^ (*Lepidozona mertensii*) to 0.1 mm^3^ (*Rhyssoplax polita*) (Fig. 4b). Despite size differences, a majority of the scales exhibited the same simple hook-like form, except *L. mertensii* (*Ischnochitonidae*), which displayed a unique geometry with two inflection points (Figs. 4e and 5d). While in most species the prismatic base accounted for ca. 30–40% of the scale height (i.e., *h*_1_/*H*: ca. 0.3–0.4), the prismatic base was ca. 50% of the total height in *Chiton cumingsii* and *Ischnochiton contractus*. The imbrication angle, *α*, ranged from ca. 90° in *I. australis* and *L. mertensii* to ca. 130° in *I. lentiginosus*. The inclination angle, *β*, ranged broadly from ca. 60° in *I. australis* to ca. 10° in *C. cumingsii*. In all species, the sum of the imbrication and inclination angles was <180°, signifying that all scales were capable of overlapping with their neighbors. *β* was usually larger in the *Ischnochitonidae* than in the *Chitonidae*, and as such, scales from the *Ischnochitonidae* exhibit a greater degree of inclination along the posterior direction for the diamond-shaped prismatic ventral base. A general morphological difference between the two examined groups was that Ischnochitonid scales exhibited more pronounced ridge structures on their dorsal surfaces. The *H/L*, *W/L*, and *A*_1_*/A*_total_ ratios for all scales ranged from ca. 0.4–0.8, ca. 0.4–0.8, and ca. 0.1–0.6, respectively.

### Parametric modeling of chiton girdle scales

We developed a parametric geometrical model to reproduce the observed morphometrics of chiton scales to facilitate the subsequent modeling of chiton-inspired scaled arrays. First, three principal sections were made through the scale: one horizontal section through the base (denoted as BASE) and two vertical sections running transversely and longitudinally in the (YZ) and (XZ) planes, respectively, both passing through the geometric center of the scale (Fig. 5a, top). We then selected 20 spatial markers on these principal sections. Using third-order polynomial interpolation, we generated a spline through each set of points, creating parametric versions of the three principal curves (Fig. 5b; see Supplementary Fig. 3 and Supplementary Table 1 for more details). We then generated a central spine running medially within the YZ cross-section (Fig. 5a, bottom, red line). Constructing planes normal to the spine along its length permitted the reconstruction of the complete scale surface. Parametrically controlling the relative positions of spatial markers along the principle curves enabled variation of scale geometries across a large design space (see Supplementary Video). Figure 5c illustrates the modeled geometry for *R. canariensis*, which agrees well with the geometry obtained from the native *μ-CT* data. The doubly curved geometry that characterizes the *L. mertensii* scales could also be replicated using the same parametric model (Fig. 5d), highlighting the morphological diversity that can be accommodated with this approach.

**Fig. 6:**
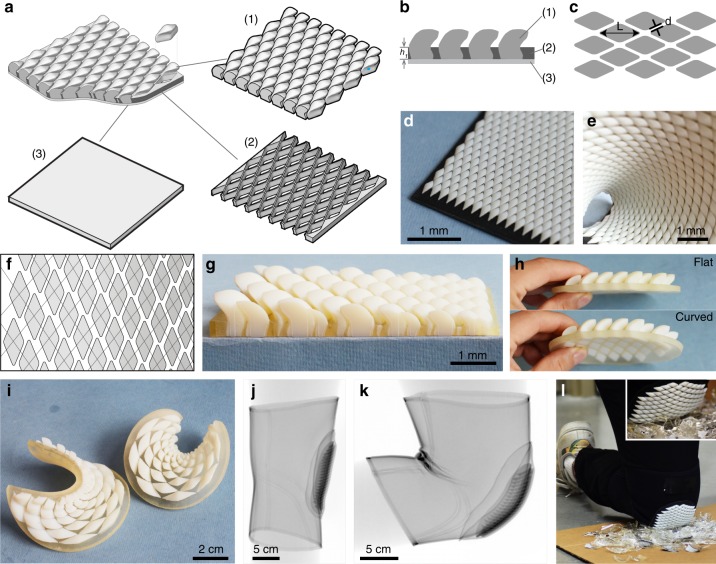
Design and fabrication of a bio-inspired flexible scaled armor. **a** Schematic diagram showing the basic components of the bio-inspired scaled armor, i.e., (1) scales, (2) matrix, and (3) soft underlying layer. **b** Side- and **c** bottom view of the scaled armor. *d*, the inter-scale spacing. **d** Flat panel with uniform scales fabricated through additive manufacturing. **e** A bent panel showing its excellent flexibility. **f**, **g** Design of scale pattern with size gradients. **h** Scale assembly in flat (top) and curved (bottom) substrate. **i** Scale assembly on double-curved surfaces. **j** X-ray projection images of a kneepad based on the bio-inspired scaled protective panel in **j** extended and **k** bent positions, demonstrating its conformability and flexibility. **l** Demonstration of the protection capability of the chiton scale-inspired kneepad on broken glass.

The successful 3D modeling of individual scales allowed us to design a composite scale armor assembly similar to that of chitons. The bio-inspired armor system included rigid scales embedded in an underlying soft substrate (Fig. 6a). The surrounding substrate matrix was modeled with a thickness equal to the height of the inflection point of the scale (*h*_1_), as in *R. canariensis* (Fig. 6b). The ratio between the inter-scale spacing *d* and scale length *L* (*d/L*, ca. 5%) was also similar to that in the native girdle (Fig. 6c). To create a uniform pattern, scales with the same size and geometry were arranged into a diamond grid shown in Fig. 6c.

Prototypes of bio-inspired scale armor were fabricated using multi-material 3D printing (Fig. 6d–l). To mimic the interaction of scales and soft girdle tissue, materials with moduli of ca. 2 GPa and ca. 0.7 MPa were used for the scales and surrounding matrix, respectively. Owing to the manufacturability and material choice limitations, the design was simplified to include only the large dorsal scales and a homogeneous flexible basal layer. The resultant fabricated scale assembly exhibited excellent flexibility, with ranges of motion similar to its biological analogue (Fig. 6e). The parametric nature of our model allowed us to quickly and efficiently explore arrangements beyond uniform patterns on a flat substrate. For example, we first explored varying the scale size in a fashion similar to that of the chiton’s native girdle scale geometry. Rather than using two sets of intersecting parallel lines as was done for the construction of the uniform scale tiling shown in Fig. 6a–d, we instead used the intersection points of two expanding fan-like grids to dictate the scale positions and sizes needed to create a gradual change in scale size (Fig. 6f, g), which closely resembled the geometry of the native girdle scales (Fig. 2b). In addition to flat substrates, substrates of varying curvature were also explored. Figure 6h shows a comparison between flat and low-curvature assemblies of scales with uniform sizes. Figure 6i shows a high-curvature assembly, where local surface curvature dictates both the orientation and size of each individual scale.

Finally, to demonstrate the utility of the chiton-inspired system for applications requiring both combined flexibility and protection, a scaled kneepad prototype was developed (Fig. 6j–l). Current kneepad designs often fall in one of two extremes: hard and rigid plates that create heavy protection but limit flexibility, or elastomeric rubbers/foams that provide high flexibility but limited protection (especially against sharp objects). The chiton scale-inspired knee protection pad offers a unique solution to this dilemma. The printed scale assemblies were easily attached on or inserted into standard knee sleeves and exhibited good shape conforming capabilities in both bent and extended configurations (Fig. 6j, k). The system also provides much higher puncture resistance to hard sharp objects compared to typical kneepads with single-material rubber- or foam-based inserts, as illustrated in Fig. 6l and insets.

### Anisotropic flexibility

**Fig. 7:**
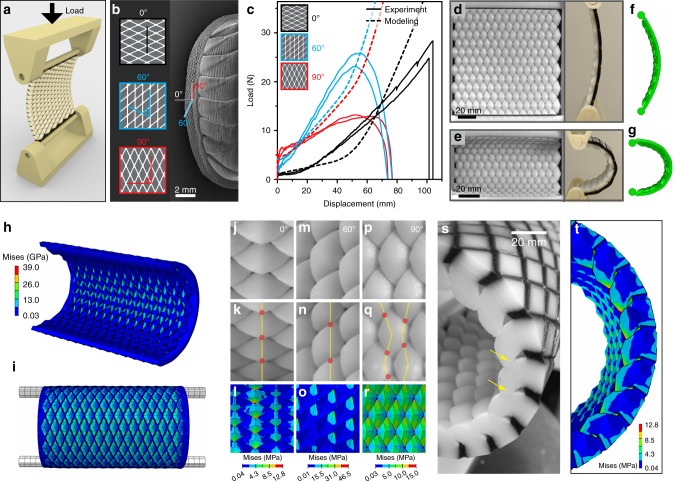
Analysis of anisotropic flexibility of the bio-inspired scale armor prototypes. **a** Schematic diagram of the post-buckling bending experiments. **b** Left, schematic diagrams of specimens tested in three orientations by varying the angle, *φ*, which is defined between the loading direction and the width direction of scales. Right, the modeled three *φ* angles indicated on the chiton’s body. **c** Experimentally measured and corresponding FE predictions of the force–displacement curves for prototypes with *φ* of 0°, 60°, and 90°. Photos of the *φ* = 90° sample at displacement of **d** 0 and **e** 60 mm, and **f**, **g** corresponding configurations from the FE modeling. The left and right photos in **d**, **e** show the front and side views, respectively. The distributions of von Mises stress of the *φ* = 90° sample from **h** the front and **i** the back view. Magnified views of the interaction between adjacent scales at displacement of **j**, **m**, **p** 0 and **k**, **n**, **q** 60 mm for *φ* = (**j**, **k**) 0°, (**m**, **n**) 60°, and (**p**, **q**) 90° sample, respectively. The red dots indicate the interlocking (contact) among adjacent scales. The von Mises stress distributions within individual scales at displacement of 60 mm due to interscale contact for *φ* = (**l**) 0°, (**o**) 60°, and (**r**) 90° sample, respectively. **s** A magnified view of the interlocked scales (indicated by yellow arrows) and **t** corresponding stress distributions from FE analysis for the *φ* = 0° sample at displacement of 60 mm.

Owing to the anisotropic geometry of the scales, we hypothesized that the scale assembly would exhibit orientation-dependent bending stiffness. Using the scales of *R. canariensis* as a model system, our parametric prototypes allowed us to systematically study the mechanical performance of the chiton scale assembly. Using multi-material 3D-printed models, we were able to conduct direct mechanical tests on these prototypes using a pinned post-buckling bending test (see section Mechanical testing of 3D-printed protypes in Methods). Each prototype was printed with rigid rods attached to either side, which were inserted into two supporting bases that were connected to the mechanical testing rig (Fig. 7a). The pinned boundary conditions defined the axis of displacement and allowed the pins to rotate freely about their central axes during the bending tests. For these measurements, we only considered concave bending modes (scales facing inwards), so that we could directly investigate the mechanical consequences of scale jamming and other scale–scale interactions. This is in contrast to the convex bending mode, which was not constrained due to the lack of scale–scale interactions and therefore any mechanical properties would be largely dependent on the flexibility of the inter-scale flexible phase. We defined the scale orientation angle, *φ*, as the angle between the loading axis of the prototype and the overlapping (hook) direction of the scales (Fig. 7b). Three prototypes were fabricated (*φ* = 0°, 60°, and 90°), where the 0° sample corresponds to a bending of the scale array towards the chiton’s body, and the 90° sample models anterior-posterior bending of the girdle.

The force–displacement curves from the bending tests are summarized in Fig. 7c, and finite element (FE) analysis based on models with same number of scales under the same loading conditions was also conducted to investigate the deformation process at both the local scale and the global assembly level (Fig. 7d–g). Figure 7d, e depict a 90° sample at the initial and later stage of the test, whose deformation behavior was also successfully captured in the simulations (Fig. 7f, g). The stress distribution maps indicate that the central region of the test samples has an overall high stress level due to its smaller local radius of curvature compared to the two edge regions (Fig. 7h, i). The predicted orientation-dependent mechanical behavior was observed from the force–displacement curves (Fig. 7c). In general, all three orientations demonstrate a certain degree of strain stiffening behavior, although the onset and rate of stiffening varied significantly. The initial soft regime with low bending resistance corresponds to the scales rotating relative to one another. The subsequent strain stiffening is a function of adjacent scales coming into contact with one another, with the differing strain stiffening behaviors the result of the different scale interactions modeled. Since the stiffening rate is governed by post-contact friction behavior, the lack of measured surface friction properties for the 3D-printed models inadvertently leads to the discrepancy between experimental and computational load-displacement curves in the post-contact regimes (Fig. 7c). The system is highly flexible in the 0° orientation (bending toward the chiton’s body) as exhibited by its lower initial bending load and larger soft regime compared to the other two orientations. In this orientation, and when viewed dorsally, the hook of each scale contacts the back of the scale immediately in front of it (Fig. 7j–l), which was confirmed in sectioned profile views both experimentally (Fig. 7s) and computationally (Fig. 7t). In the cases of the 60° and 90° samples, each scale eventually made contact with neighboring scales in the vertical and adjacent diagonal rows, respectively (Fig. 7m–o and Fig. 7p–r, respectively). For the 90° sample, despite early contact and four contact points per scale (Fig. 7q, red dots), the scales are able to slide past each other with relative ease as indicated by the lower stress levels within scales (Fig. 7r), resulting in a low stiffening rate. In contrast, for the 60° samples, the scales make contact with adjacent scales in a single row (Fig. 7n); however, a much higher local contact stress due to significant interlocking among adjacent scales (Fig. 7o) leads to a higher stiffening rate.

## Discussion

Similar to other biological flexible armor systems, such as the imbricated scales of fish and the osteoderms of reptiles, the chiton species highlighted here also utilize tiled subunits to achieve both protection and flexibility^5^. The chiton girdle scales, however, exhibit a significantly different design in terms of material, interfacial, geometrical, and mechanical characteristics compared to fish scales or osteoderms. Chiton scales have a very homogeneous and high mineral content (ca. 97 wt%)^43^ and are composed of tightly packed, aragonitic, nano-scale rod-like building blocks. Although the ganoid scales of some fishes (e.g., members of the Polypteriformes) possess an enamel-like outer layer (ganoine), which can have a high mineral content (>90 wt%)^55^, the main structural component of these scales is bone, which typically has a mineral content of ca. 65% by weight^56^. The bone in osteoderms has a similar mineral content^19,21^. As a result, and to the best of our knowledge, the girdle scales of chitons are among the most heavily mineralized of all known natural scale-based armors^1,5^. Moreover, unlike most fish scales and osteoderms^5^, chiton scales are comparatively more uniform in composition, exhibiting no sub-layering, material heterogeneity, or porosity. This observation underlines the suitability of chiton scales as a model for bioinspiration, as the mechanical performance of their armor can be ascribed primarily to geometric considerations, rather than fine scale material variation.

In contrast to the quasi-two dimensional geometries of many fish scales, chiton scales are truly three-dimensional structures. However, their mechanism of structural interlocking, a function of their hook-like morphology, is different compared to that in osteoderms, which involve complex, fingerlike interdigitations. Chiton scales are characterized by a single curvature structure consisting of two parts, i.e., a prismatic base and a hook-like upper region that overlaps with neighboring scales. The base of each scale has a diamond-shaped cross-section and is usually inclined backwards, by *β* = 10–60°. The cross-sectional geometry of the base does not change along its height, and the hook-like region of the scale curves forward, resulting in an imbrication angle *α* ranging from ca. 90° to ca. 130°. The length ratios of the scales were generally consistent (with some slight variations) among the ten species investigated, and the center of mass of each scale type lies within the basal region, suggesting an intrinsic resistance to tilting under normal compressive loading conditions (Supplementary Fig. 4). This resistance to tilting is also beneficial when the scale armor under a predatory attack-like concentrated loading. As shown in Supplementary Fig. 5, during a puncture test, the indenter tends to slowly slide off the scale surface due to the scales’ curved surface. As the scales do not tilt during this process, the penetration resistance and puncture force gradually increase due to the interlocking effect between the indenter and contacting scales, in contrast to the scale armor in gar fish^57^.

While geometrically the girdle scales of chitons bear a striking resemblance to the placoid scales of some shark species, which also possess a diamond-shaped base and a dorsal, overlapping hook-like cusp/crown^58^, these structures have evolved independently in two taxa. Any observed similarities are likely related to their shared protective function. Interestingly, the variation in the degree of overlap observed between neighboring chiton scales, characterized by the ratio *A*_1_*/A*_total_ (0.1–0.6), is similar to that seen in fish scales (0–0.76)^59,60^. One clear advantage of overlapping scales in armors is that they permit movement of individual scales, while still allowing effective coverage of inter-scale regions, leading to uniform protection and resistance to penetration^27,61^. This protection strategy is not limited to chitons. Similar strategies are also found in the scale and plate-like armor of fish, reptiles, and mammals to protect weak interfaces from direct damage during predatory attacks^61,62^. Moreover, the chiton dorsal girdle scales are arranged such that they overlap (hook) towards the body, ensuring complete coverage of the transition region between the girdle and the eight overlapping primary dorsal shell plates.

Similar to fish and pangolin scales, chiton scale armors permit a high bending flexibility along the convex direction (i.e., forming a ventral cup toward the substrate), limited only by the elasticity of the underlying and connective soft tissues. Convex flexibility facilitates locomotion and enables animals with scaled armors to adopt extreme defensive postures like rolling into a ball^63^. In the case of chitons, the convex flexibility also allows the girdle scale armor to tightly conform to bumpy substrates, and thus plays an important role in mitigating water loss, as desiccation poses a great environmental threat for intertidal species like chitons^64,65^. In addition to convex flexibility, concave flexibility and associated scale interactions are extremely important for scaled animals to resist external loads from predatory attacks, as chitons have a variety of known predators, including birds^66^, fish^67^, crabs^68^, starfish^69^, lobsters^70^, octopuses^71^, and snails^72^, and also allow the animals to conform to depressions in rocks. In general, the mechanics of concave bending are related to the geometry of the scales and their mechanical properties. Similar to fish scales, the concave flexibility of chiton scales results from scale rotation, with scale–scale contact restricting flexibility beyond a certain point.

Our bio-inspired parametric modeling and multi-material 3D printing approach allowed us to systematically investigate the mechanical behavior of chiton scale armor. The 3D-printed chiton scale assemblies exhibited strain stiffening^56^, as characterized by their J-shaped load-deformation curves during bending tests. Whereas strain stiffening in teleost fish scales is primarily due to the bending deformation of individual scales once in contact^17^, the increased stiffness of chiton girdle armor at higher strains primarily results from scale interlocking and jamming, a result of the non-deformable nature of the ceramic scales. In both of these mechanisms, strain stiffening enhances protection by stiffening the scale array, resisting penetration and maintaining structural integrity. The increase in stiffness upon interlocking also helps redistribute an applied external load over large areas by involving more scales^17,61^.

Since strain stiffening in chiton girdle armors arises from geometric constraints, this behavior is highly dependent on scale geometry, size, and loading orientation. The strain stiffening of chiton scale armor is anisotropic due to the non-axisymmetric 3D geometry of the scales, and the bending tests of the 3D-printed models provide mechanistic insights into this behavior. Stiffening onsets earliest in the 90° orientation compared to 0° and 60°; however, the 90° orientation exhibits the lowest stiffening rate. These observations are based on scales in the 90° orientation being able to slide past one another after contact occurs, while the scales are interlocked more tightly in the 0° and 60° orientations. The 0° orientation has the largest bending capability before scale interlocking, and as such, is also the orientation with the highest flexibility. The ridges along the 0° direction observed in the dorsal surfaces of scales, especially in members of *Ischnochitonidae*, may function to increase friction between scales in contact, resulting in an increased strain stiffening response, especially in the 90° orientation. Anisotropic flexibility of other scaled armor systems is also achieved via joints (e.g., the peg and socket structure of some ganoid scales^15^) and anisotropic scale deflection and rotation (e.g., in teleost fish)^14,27^. In the tessellated cartilage of sharks and rays, it is hypothesized that the anisotropic geometry of individual tesserae and complex joint surfaces may lead to an anisotropic mechanical behavior^24^. Biological tiled architectures therefore offer a myriad of models for the design of bio-inspired surfaces, constructed from relatively simple subunits, but with tunable, orientation-dependent stiffness.

The local balance of flexibility and protection in the girdles of chitons is primarily controlled through the variation of scale size. For example, at the distal margin of the chiton girdle, a relatively high degree of flexibility is needed to conform to rough surfaces, so small scales are employed. In contrast, at the proximal margin near the body of the chiton, a high degree of protection is required, so large, thick scales are present. Similar strategies for regional regulation of penetration resistance and flexibility through control of local scale size, geometry, and assembly pattern have been also reported in other biological scaled armor systems^15,16^. Ancient Scythians were aware of this body armor design principle well over 2000 years ago and used small scales in areas of the armor intended to bend (e.g., elbows and shoulders), and large scales in vital areas of the torso for better protection^73^. The manufacturers of Japanese samurai armor also followed similar design concepts and made use of several sizes of protective scales, sometimes arranged in gradients, to construct different protective elements^74^. Understanding the geometric and material design principles of natural scale armor systems, like that of chiton girdle scales, thus enriches the design space for the next generation of armor development. In this process, computational design tools and additive manufacturing technologies will continue to enable rapid progress from armor concept to functional structure production^15,16,28–31^.

## Methods

### Samples

Entire dried shells of *Rhyssoplax canariensis* and *Chiton cumingsii* were purchased from commercial sources. Most other samples were 70% ethanol-preserved specimens from the collection of D.E., including *Rhyssoplax jugosa*, *Rhyssoplax polita*, *Radsia barnesii*, *Ischnochiton* (*Heterozona*) *fruticosus*, *Ischnochiton* (*Ischnoradsia*) *australis*, *Ischnochiton* (*Haloplax*) *lentiginosus*, and *Ischnochiton* (*Ischnochiton*) *contractus*, and *Lepidozona mertensii*.

### Electron microscopy

Intact girdle fragments and individual scales from the chiton *R. canariensis* were coated with either ultra-thin carbon or Pt/Pd prior to observation with SEM. Samples were imaged using a Helios Nanolab 600 or 660 Dual Beam electron microscope (FEI, OR) at an acceleration voltage of 2 kV and a working distance of ca. 4 mm. EDX was performed on polished cross sections with a Helios Nanolab 600 Dual Beam (FEI, OR) equipped with an INCA EDX system (Oxford Instruments) at an accelerating voltage of 20 kV. Whole-animal SEM imaging was performed with a Tescan Vega GMU scanning electron microscope (Brno, Czech Republic) at 20 kV. Three-dimensional reconstruction of the fracture surfaces was achieved by first taking stereo SEM image pairs with the sample stage tilted at 10° and −10°, then analyzing and rendering with MountainsMap® imaging analysis software.

### Synchrotron-based µ-CT

Girdle samples from all of the chiton specimens were scanned with an energy of 20.4–23.8 kV and a resolution of 2.84 μm per voxel at beamline 2-BM of the Advanced Photon Source at Argonne National Laboratory. Image processing software package Mimics (Version 15.0, Materialize, Belgium) was used for segmentation and construction of 3D triangulated surface meshes (binary stereolithography (STL) format). The dorsal scales used in the morphometric study were selected from the middle (between the proximal and distal margins) of the girdles. The scales were segmented using a threshold brush technique, which allows the user to highlight pixels over a specific grayscale range in an area defined by the b`rush location, size, and shape. Additional manual segmentation was limited to the removal of ring artifacts. To create scale armor models, the surface meshes of the scales were first reduced in file size as needed via the smooth, reduce, and remesh functions of 3-matic (Materialize, Belgium). Cross-sectional meshes were created using the cut function of the simulation module Mimics. Open source image analysis software ImageJ was used to identify landmarks and make measurements from 2D cross-sectional images, and 3D rendering of individual scales and scale assemblies were performed using Blender (Blender.org).

### X-ray imaging of bio-inspired flexible protective panel

To visualize the bending of the different components of the kneepad prototype, the 3D-printed chiton scale construct was sewn into a neoprene sleeve, which was then placed on either an extended or bent CNC-milled Styrofoam knee joint model. Each of these models was then imaged in transmission using an XRA-002 × -Tek micro-CT system (Nikon Metrology, Brighton, MI, USA) for data acquisition.

### Instrumented nanoindentation

Girdle samples of *R. canariensis* were embedded in a room temperature curing epoxy. After curing for ca. 24 h, samples were sectioned with a diamond saw (IsoMet 5000, Buehler, Lake Bluff, IL), polished (Model 920, South Bay Technology, CA) stepwise with aluminum oxide pads (15, 5, and 3 μm), and then finely polished with 50 nm silica nanoparticles on cloth pads (MultiTex, South Bay Technology, CA). Nanoindentation experiments were conducted in ambient conditions using a TriboIndenter (Hysitron, Inc., Minnesota, USA). Load-controlled nanoindentation was performed using Berkvoich (trigonal pyramid, semi-angle = 65.3°) diamond probe tips. The piezoelectric transducer was first allowed to equilibrate for 105 s (the last 45 s with digital feedback) and another 40 s for calculating drift automatically prior to each indent. Typical load functions included loading (10 s), holding (20 s), and unloading (10 s), with maximum load of 2 mN. The standard Oliver-Pharr (O-P) methodology was used to quantify material properties, i.e., indentation modulus (*E*_*O-P*_) and hardness (*H*_*O-P*_). The probe tip area function *A*(*h*_c_), which is the projected area of the indentation tip as a function of the contact depth *h*_c_, and frame compliance were calibrated prior to each set of experiments using a fused quartz sample. Unfortunately, due to its resin-embedded nature and uneven polished surface at the middle fibrous layer, it was not possible to accurately measure the mechanical properties of the fibrous organic region.

### Parametric modeling

The iso-surface of *R. canariensis* scales based on the volumetric µ-CT data was first selected for initial parametric modeling, which was then extended to scale geometries from other species. NURBS/CAD modeling software Rhinocores® (Robert McNeel & Associates) and its parametric modeling plugin Grasshopper® were used to generate the parametric models. Three sectional cuts (BASE, YZ, and XZ) were first extracted and markers were drawn along its length—the minimum number of points representing the main curvature variations of the sectional outline. More specifically, the BASE section was a diamond-shaped geometry with four parameters, including width, length, and fillet radii. The YZ section was the most representative of the scale geometry and included ten parameters representing 7 points’ spatial coordinates. The YZ section also inherited two parameters from the BASE section. The XZ section included three parameters in addition to one inherited form YZ and two inherited from BASE. In total, the parametric model included 20 points that were adjustable using 17 parameters—as some parameters controlled multiple points simultaneously. Any parameter value change would propagate through the entire model thus creating a new iteration. With the sectional geometric scaffold in place, the chiton scale surface was to be generated. A central spine was extracted medially within the (YZ) curve as shown in Supplementary Fig. 3. Equally spaced perpendicular planes were generated along the spine. Intersections between (YZ/XZ) sections and these planes were resolved. A curve was then interpolated through the resultant four intersection points at each plane. Multiple curves were lofted creating the chiton scale solid model. The parametric scale model was also tested to generate other scale geometries, including the double-curved scale from the chiton *Lepidozona mertensii*. After the individual scale geometries were modeled, 3D models of scale armor assemblies were created using 3D modeling software Rhinoceros®. Each armor model consisted of two components, an array of scales and a flat underlying layer in which the scales were embedded. A number of scale arrangement patterns were tested, including uniform diamond pattern, gradient in sizes, and patterning on curved surfaces (see Supplementary Movie).

### Three-dimensional printing

Tangible prototypes were fabricated on a Connex500 multi-material 3D printer (Stratasys, Eden Prairie, Minnesota, USA) by using STL files of both scales and substrate generated from the chiton-inspired scaled armor design. At each print layer, flexible, and rigid material photopolymers of different colors were simultaneously ejected and eventually UV-cured into a single build. The material used for the scales were the rigid white VeroWhitePlus® (RGD835), while those used for the substrate were either the flexible black TangoBlackPlus® (FLX980) or the flexible transparent TangoPlus® (FLX930). While the two flexible polymers exhibited similar properties, the use of the two different colors varied as a function of their intended application, with the clear flexible material being employed in the earlier models in order to more easily detect possible design and fabrication defects such as model voids, or poor scale-substrate adhesion. Once these fabrication issues had been resolved, all subsequent models (and those used for mechanical characterization) were printed with flexible black material in order to exhibit better contrast when photographed. The two 3D-printed materials employed in the production of the scales and substrate, respectively, represented the extremes in mechanical properties available for use with our employed 3D printer, with the Vero family being the stiffest (with a modulus of ca. 2.0 GPa) and Tango family the most flexible (with a modulus of ca. 0.7 MPa). While the employed printer is capable of altering the mixing ratio of these two extremes to produce other materials with hybrid mechanical properties, this study only focused on utilizing the source materials, which represented an ca. 1000-fold in modulus difference. This decision was chosen in order to more clearly characterize the effects of geometry on the mechanical performance in the 3D-printed composites, while approximating qualitative girdle tissue mechanical properties derived from studies of living chitons^75^ and in-situ field observations by the coauthors.

### Mechanical testing of 3D-printed prototypes

The 3D-printed chiton girdle-inspired mechanical test specimens were designed and directly printed with two pins (printed with VeroWhitePlus) at either side, which ensured the pinned boundary condition during testing. A customized sample holder was designed to fit around the rigid pins and conduct the post-buckling bending test. Each prototype had a span and width of 124 mm and 112 mm, respectively. The individual scale length, *L*, and inter-scale spacing distance, *d*, of the prototypes were 10 mm and 0.5 mm, respectively. The embedded height of the scales, *h*_1_, which was also the thickness of the TangoBlack substrate, was 4.1 mm. Three types of prototypes with different scale orientation angle (*φ* = 0°, 60°, and 90°) were fabricated, and were cleaned with a water jet and air dried before testing. The prototypes were experimentally tested in bending induced by axial compression on a mechanical tester (Zwick Z010, Zwick Roell, Gemany) using load cells ranging from 20–2500 N. Sample holders were fixed to the load cell with Permacel (Nitto) tape for pin–pin boundary conditions that allowed rod rotation about the *x*-axis and constrain rotation about the *y*- and *z*-axes to prevent global twisting of the sample. The samples were subjected to post-buckling bending deformation concavely (scales facing inwards) by setting an initial lateral deflection of 10 mm and zeroing the force before displacement-controlled compressive loading at a displacement rate of 1 mm s^−1^. The reaction force vs. vertical displacement for the prototypes was measured, and the experiments were performed with two samples (*N* = 2) along each orientation, which demonstrated good repeatability for each test condition. Photographs of the specimens during tests were acquired at a rate of 0.5 fps (VicSnap, Correlated Solutions).

### FE analysis

A computational framework was established for simulating bending of the chiton-inspired flexible armors. For each simulation, three parts were modeled using finite element software (ABAQUS): scales, a substrate, and two rigid rods. A simplified scale geometry was utilized to model the scales. For the substrate, a 120 mm × 124 mm rectangle plate with a thickness of 3.441 mm with grooves to fit the scales was used. Cylindrical rods were modeled as rigid bodies to be placed at the top and bottom of the substrate. The scales and substrate were meshed with tetrahedral elements, and all the simulations were performed for three orientation angles (*φ*) of 0°, 60°, and 90°. VeroWhite and TangoPlus were modeled as an isotropic linear elastic material assigned to the scales, and a Neohookian hyperelastic material was assigned to the substrate, respectively^76–78^. In order to assemble the parts, the scales were placed in the grooves of the substrate and tied to the grooves. The rigid rods were also tied to the top and bottom of the substrate. Hard contact (surface to surface) was defined between the surfaces of the scales, which might be in contact due to the loading deflection. For simulating the bending, pin–pin boundary conditions allowed rotation of the rods about the *x*-axis and translation of the top rod in the *y*-axis were used to constrain the model. During evaluation, three steps were applied to the structure: (1) rotation of both rods to set a concave bending with a lateral deflection of 10 mm, with free translation of the top rod, (2) relaxation allowing free rotation of the rods with fixed displacement of the top rod to relieve internal stresses, and (3) translation (−*y*) of the top rod with a velocity of 1 mm s^−1^ and with free rotation of the rods to bend the model. Force–displacement curves were calculated as a measure of the reaction force vs. vertical displacement of the top rod, and von Mises stresses were captured through the whole model to show the stress distribution during loading.

## Supplementary information

Supplementary Information

Supplementary Movie 1

Description of Additional Supplementary Files

## Data Availability

Data generated from this study are included in this article and its Supplementary Information files or will be provided from the corresponding author upon reasonable request.
