# Supplementary material for: Bioinspired design of flexible armor based on chiton scales
Source: Nat Commun. 2019 Dec 10;10:5413. doi: 10.1038/s41467-019-13215-0 (PMC6904579; doi:10.1038/s41467-019-13215-0)
Supplement: Supplementary file 1 — Supplementary Information [file 41467_2019_13215_MOESM1_ESM.pdf]

Supporting Information

for

Bioinspired design of flexible armour based on chiton scales

Matthew Connors[1]*, Ting Yang[2]*, Ahmed Hosny[3], Zhifei Deng[2], Fatemeh Yazdandoost[2], Hajar Massaadi[1], Douglas Eernisse[4], Reza Mirzaeifar[2], Mason N. Dean[5], James C. Weaver[6], Christine Ortiz[1], Ling Li[2]^

[1]Department of Materials Science and Engineering, Massachusetts Institute of Technology, Cambridge, MA 02139-4307, USA.
[2]Department of Mechanical Engineering, Virginia Polytechnic Institute and State University, Blacksburg, VA 24060, USA.
[3]Department of Radiation Oncology, Dana-Farber Cancer Institute, Harvard Medical School, Boston, MA 02115, USA.
[4]Department of Biological Science, California State University Fullerton, Fullerton, CA 92834, USA.
[5]Department of Biomaterials, Max Planck Institute of Colloids and Interfaces, Am Muehlenberg 1, 14424 Potsdam, Germany.
[6]Wyss Institute for Biologically Inspired Engineering, Harvard University, Cambridge, MA 02138, USA.

* Equal contributions.
^ To whom correspondence should be addressed. E-mail: lingl@vt.edu

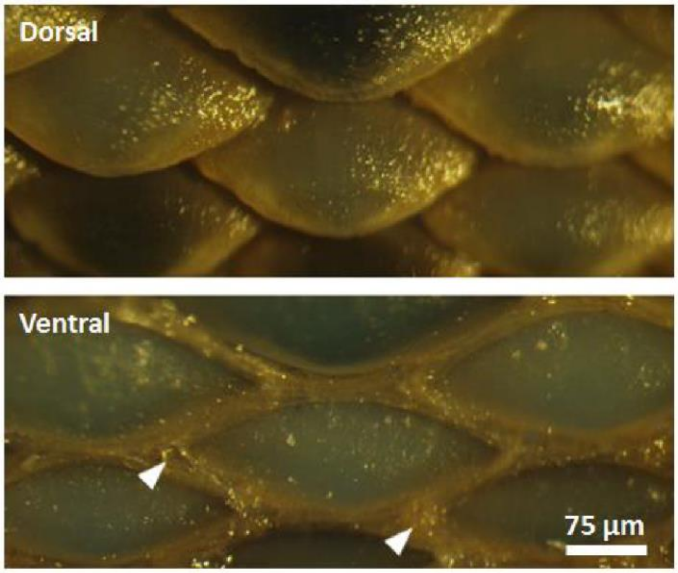

Supplementary Figure 1: Light micrographs of the dorsal girdle scales of *Chiton sp.* after removing the underlying soft tissue of the girdle. Note that in the ventral (lower) image, organic material (indicated by white triangles) is present between the scales, robustly connecting them together.

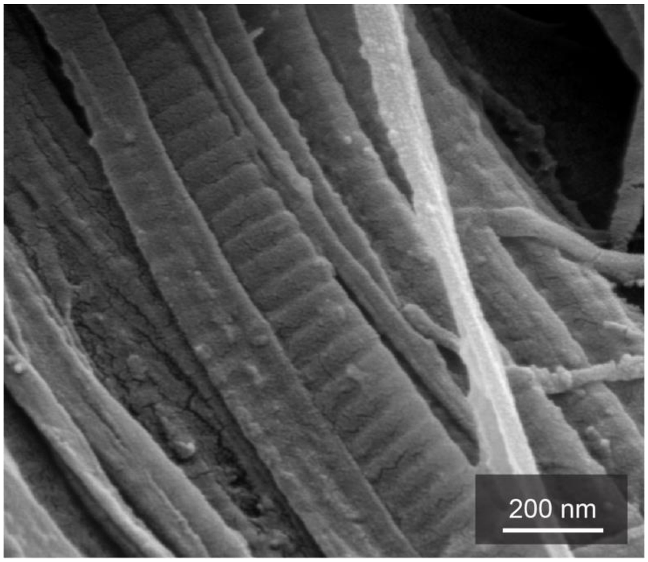

Supplementary Figure 2: Banded, collagen-like filaments observed in the fibrous middle layer of the girdle from the chiton, *Rhyssoplax canariensis*.

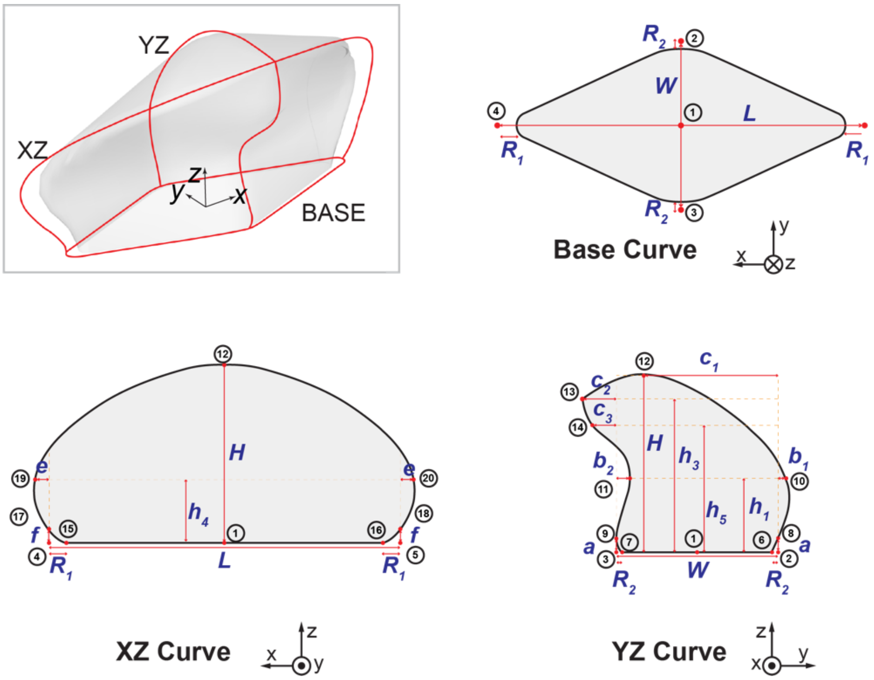

Supplementary Figure 3: Detailed schematic of the spatial markers along the principle curves of a single chiton girdle scale and relevant dimensional parameters.

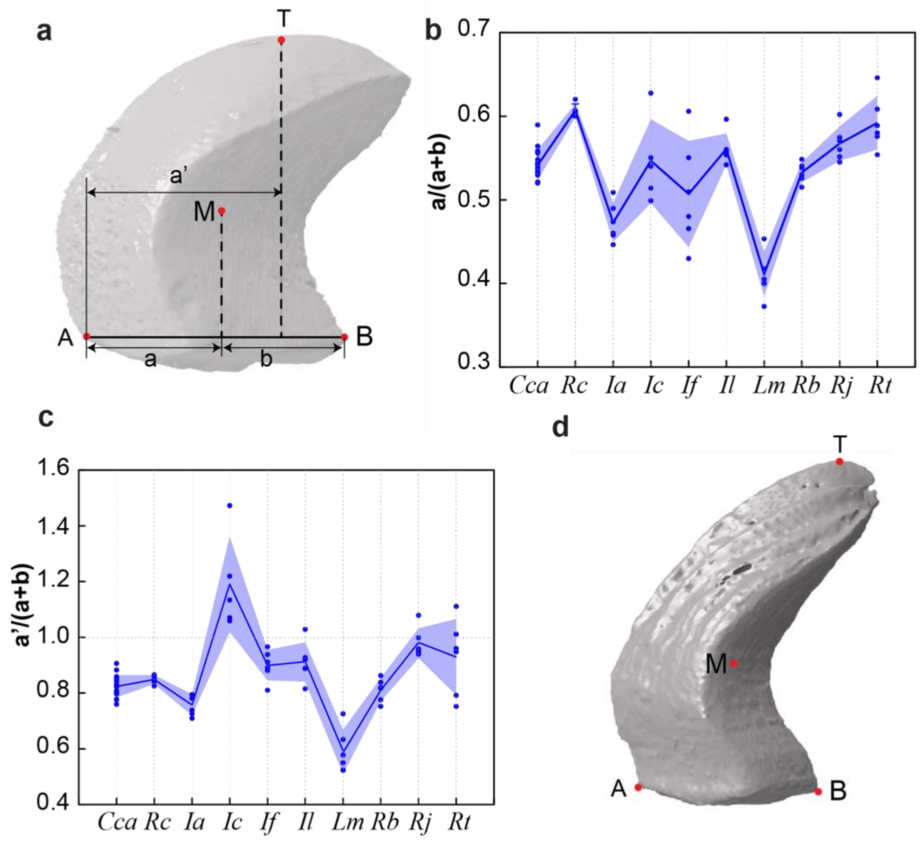

Supplementary Figure 4: Analysis of potential scale tilting of individual scales. **a**. Side view of a representative scale from *R. canariensis*. Point ***A*** and ***B*** represent the two edge points. ***M*** is the center of mass and ***T*** is the highest point of the scale. The relative positions of ***M*** and ***T*** are measured by ***a/(a+b)*** and ***a'/(a+b)***, where ***a***, ***b***, and ***a'*** represent the lengths in the diagram. The distribution of relative positions of (**b**) ***M*** and (**c**) for different species. The solid line and shaded area represent average and standard deviations (n = 6). Note that the measured values of ***a/(a+b)*** range from 0.4-0.6, which indicates that the three-dimensional scales will not tilt under its own weight. The measured values of ***a'/(a+b)*** are also generally smaller than 1 except for *I. contractus*, which indicates that these scales will not tilt under normal compression loading at the highest point of the scales. **d**. Illustration of relative positions of ***M*** and ***T*** for *I. contractus*.

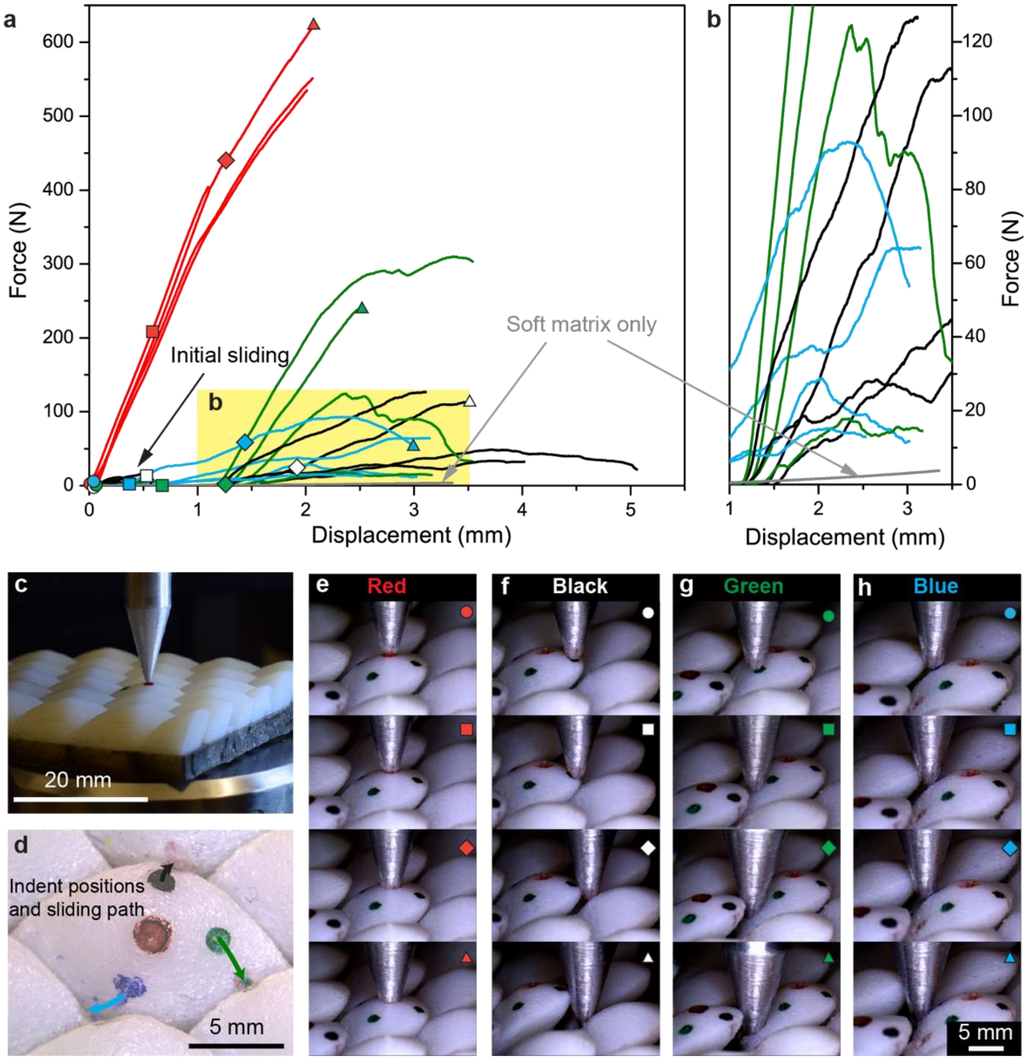

Supplementary Figure 5: Puncture test on bio-inspired printed scales. **(a)** Load-displacement curves for tests on printed scales at four different positions of individual scales: center (red point), close to front end (black point), point close to the side end (green point), and close to rear end (blue point) (see (**c**)). **(b)** Magnified region in the load-displacement curves highlighting the increase in penetration resistance after the indenter slides off the scales. **(c)** Test setup. **(d)** Test positions colored corresponding to the load-displacement curves in **(a)**. **(e-h)** Sequence of images of the puncture tests at different displacements (indicated by the corresponding labels in **(a)**). These measurements show that the puncture on the center points behaves as a steady indentation with local deformation, while tests on the other locations induced sliding and subsequent scale interlocking. For indentations on the front ends (black points), the tip slid onto the adjacent scale, and the load gradually built up as the tip indented into the scale. For indentations on the side ends (green points), the initial sliding stage was further extended probably due to the larger sliding path, and when the indenter tip was transversally inhibited by adjacent side scale and indented into the scale, the load started to build up. Lastly, for indentations on the rear ends (blue points), the hook of the adjacent scale soon produced an interlocking effect that added inhibiting force to the sliding tip, avoiding tip penetrating into substrate materials. These tests indicate that the puncture-like external attack on chiton scale armors will not result in immediate indentation on the underlying soft substrate after the indenter slides off scales. Instead, the geometrical design and

organization pattern of the scales lead to the interlocking of scales and the indenter, resulting in increase in puncture forces.

Marker No	BASE Curve			YZ Curve			XZ Curve		
	x	y	z	x	y	z	x	y	z
1	x0	y0	z0	x0	y0	z0	x0	y0	z0
2	x0	y0+W/2	z0	x0	y0+W/2	z0			
3	x0	y0-W/2	z0	x0	y0-W/2	z0			
4	x0+L/2	y0	z0				x0+L/2	y0	z0
5	x0-L/2	y0	z0				x0-L/2	y0	z0
6				x0	y0+W/2-R2	z0			
7				x0	y0-W/2+R2	z0			
8				x0	y0+W/2	z0+a			
9				x0	y0-W/2	z0+a			
10				x0	y0+W/2+b1	z0+h1			
11				x0	y0-W/2+b2	z0+h1			
12				x0	y0+W/2-c1	z0+H	x0	y0+W/2-c1	z0+H
13				x0	y0-W/2-c2	z0+h3			
14				x0	y0-W/2-c3	z0+h5			
15							x0+L/2-R1	y0	z0
16							x0-L/2+R1	y0	z0
17							x0+L/2	y0	z0+f
18							x0-L/2	y0	z0+f
19							x0+L/2+e	y0	z0+h4
20							x0-L/2-e	y0	z0+h4

Supplementary Table 1: Coordinates of the spatial markers used in the construction of our chiton scale parametric model. See Supplementary Figure 3 for the marker positions.
