# Supplementary material for: Bioinspired design of flexible armor based on chiton scales
Source: Nat Commun. 2019 Dec 10;10:5413. doi: 10.1038/s41467-019-13215-0 (PMC6904579; doi:10.1038/s41467-019-13215-0)
Supplement: Supplementary file 3 — Description of Additional Supplementary Files [file 41467_2019_13215_MOESM3_ESM.doc]

**Title:** Supplementary Movie 1.

**Description:** Parametric model of chiton scale-inspired scale geometries.
